# Wide-field optical mapping of neural activity and brain haemodynamics: considerations and novel approaches

**DOI:** 10.1098/rstb.2015.0360

**Published:** 2016-10-05

**Authors:** Ying Ma, Mohammed A. Shaik, Sharon H. Kim, Mariel G. Kozberg, David N. Thibodeaux, Hanzhi T. Zhao, Hang Yu, Elizabeth M. C. Hillman

**Affiliations:** 1 Laboratory for Functional Optical Imaging, Department of Biomedical Engineering and Radiology, Columbia University, New York, NY 10027, USA; 2 Mortimer B. Zuckerman Mind Brain Behavior Institute, Columbia University, New York, NY 10027, USA

**Keywords:** optical imaging, haemodynamics, GCaMP, fluorescence, neurovascular coupling, spectroscopy

## Abstract

Although modern techniques such as two-photon microscopy can now provide cellular-level three-dimensional imaging of the intact living brain, the speed and fields of view of these techniques remain limited. Conversely, two-dimensional wide-field optical mapping (WFOM), a simpler technique that uses a camera to observe large areas of the exposed cortex under visible light, can detect changes in both neural activity and haemodynamics at very high speeds. Although WFOM may not provide single-neuron or capillary-level resolution, it is an attractive and accessible approach to imaging large areas of the brain in awake, behaving mammals at speeds fast enough to observe widespread neural firing events, as well as their dynamic coupling to haemodynamics. Although such wide-field optical imaging techniques have a long history, the advent of genetically encoded fluorophores that can report neural activity with high sensitivity, as well as modern technologies such as light emitting diodes and sensitive and high-speed digital cameras have driven renewed interest in WFOM. To facilitate the wider adoption and standardization of WFOM approaches for neuroscience and neurovascular coupling research, we provide here an overview of the basic principles of WFOM, considerations for implementation of wide-field fluorescence imaging of neural activity, spectroscopic analysis and interpretation of results.

This article is part of the themed issue ‘Interpreting BOLD: a dialogue between cognitive and cellular neuroscience’.

## 1. Introduction

Wide-field optical imaging of neural activity was first demonstrated 30 years ago using cortical application of fluorescent voltage-sensitive dyes (VSDs) [1]. Around the same time, it was noted that changes in optical diffuse reflectance of the exposed cortex of the brain were visible, even without application of dyes, and these changes were termed ‘intrinsic signals’ [2]. ‘Intrinsic signal imaging’ soon became a standard technique for mapping functional domains of the cortex, for example, to guide placement of electrodes [3,4]. More detailed spectral analysis of intrinsic optical signals revealed that they could be attributed to local changes in cortical blood volume and oxygenation, and in some cases scattering [5,6]. The ability to measure brain haemodynamics made this approach a popular tool for studying neurovascular coupling; the relationship between neural activity and local changes in cortical blood flow [7–16].

Although VSDs held great promise as direct optical indicators of neural activity, the need to apply them to the exposed brain prior to imaging, their very fast response times and their very small Δ*F*/*F* ratios (typically 0.15%) made them challenging to use. However, recent improvements in exogenous and genetically encoded fluorescent indicators of neural activity such as GCaMP have enabled targeted, transgenic expression of fluorescence with much higher Δ*F*/*F* levels (exceeding 10%) [17]. Additional studies have demonstrated that the dynamics of flavoprotein fluorescence can be optically mapped in the brain of wild-type animals as an indicator of oxidative metabolism [18,19]. Such wide-field neuroimaging methods are also readily compatible with optogenetic approaches to modulate brain activity in awake animals [20]. As a result, researchers have begun to return to camera-based ‘mesoscopic’ optical recording of neural activity across the exposed cortex, further facilitated by greatly improved high-speed, sensitive camera technology [21–25].

**Figure 1.**
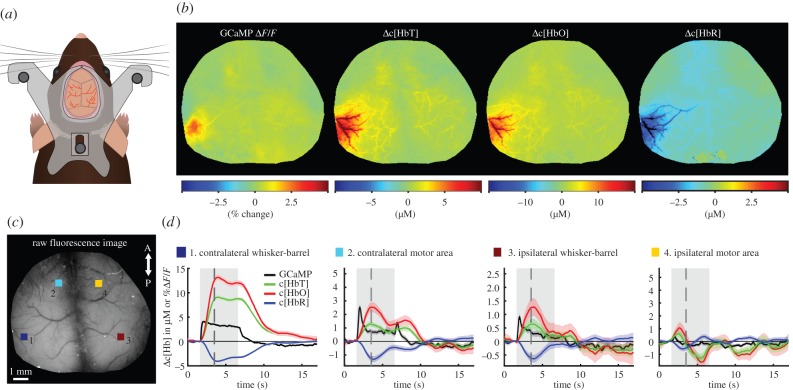
Demonstration of WFOM imaging of neural activity and haemodynamics in awake, behaving mouse brain. (*a*) A custom-made acrylic head plate is surgically implanted onto a thinned skull cranial window. After recovery, the mouse's head is held by attaching the head plate to an aluminium head plate holder to restrict head motion during imaging. The animal is free to run or rest on a saucer wheel positioned below. (*b*) Functional maps of Thy1-GCaMP6f (selectively expressed in excitatory neurons of layers 2/3 and 5 [17]) Δ*F*/*F* after haemodynamic correction, Δ[HbT], Δ[HbO] and Δ[HbR] 1.7 s after stimulus onset. (*c*) Grey scale raw fluorescence image of the cortical surface with four selected regions of interest (ROIs) in the contralateral (1) whisker-barrel and (2) motor areas, as well as in the ipsilateral (3) whisker-barrel and (4) motor areas. (*d*) Time course of neural GCaMP neural activity and haemodynamic changes in HbT, HbO and HbR corresponding to these four regions. Grey shading shows stimulus time. Black dashed line shows time of frames shown above. Data were acquired using Andor Ixon camera with 512 × 512 frames and 30 ms exposure time per frame. Data shown are an average of 38 repeated stimulation trials in one mouse, with 5 s duration, 25 Hz tactile whisker stimulation.

Here, we refer to optical imaging of reflectance (intrinsic) and fluorescence contrast in the brain collectively as ‘wide-field optical mapping’ (WFOM) of the brain. We propose that it is important to consider these two techniques as related, since similar optical principles are fundamental to understanding the constraints, resolution and depth sensitivity in both. Importantly, there is also significant potential optical cross-talk effects between detected fluorescence and dynamic changes in absorption caused by functional haemodynamics (a problem already encountered in early VSD work). It is essential that such cross-talk effects be carefully considered and accounted for in analysis to avoid misinterpretation of WFOM fluorescence data. Figure 1 shows an example of both fluorescence and haemodynamic (reflectance) WFOM, performed simultaneously in the bilaterally exposed cortex of the awake, behaving mouse brain. These data were processed using methods for converting reflectance data to haemodynamic parameters, while fluorescence recordings were corrected to compensate for haemodynamic cross-talk.

With limited commercial platforms available, home-built implementations of WFOM have varied widely between groups. Analysis and interpretation of WFOM data have thus also been varied, leading to general confusion regarding what can be understood from the method in terms of sensitivity, quantitative accuracy, depth sensitivity and optimal implementations. Recognizing WFOM's great potential as a method for mapping brain activity at high speeds over large areas of the cortex in animals ranging from mice to humans, this article provides resources for standardization of the method. The basic principles of both reflectance and fluorescence WFOM are reviewed, including approaches and considerations for analysis of data including conversion of reflectance data into haemodynamic parameters and correction of fluorescence for haemodynamic cross-talk.

## 2. Reflectance wide-field optical mapping

**Figure 2.**
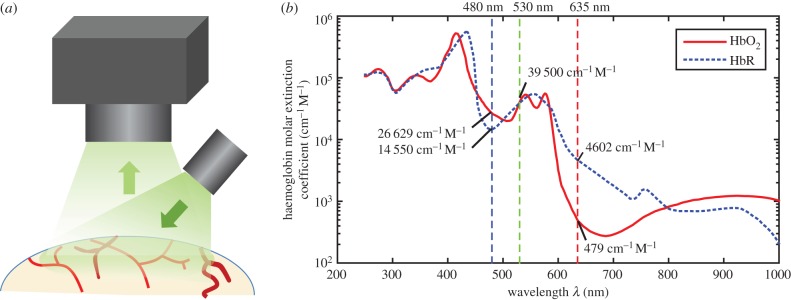
Imaging geometry for WFOM (*a*) and absorption spectra of oxy- and deoxyhaemoglobin (HbO and HbR) (*b*). Vertical lines show common wavelengths for reflectance WFOM measurements. Raw data, reproduced from [26] is tabulated in the electronic supplementary material, appendix A.

WFOM datasets are acquired using a simple camera to acquire a time sequence of two-dimensional images of the exposed surface of the brain under illumination by the light of specific wavelengths (figure 2). For reflectance WFOM, the light detected is diffusely reflected light, meaning that it entered the brain, scattered within the cortex and emerged from the same surface to reach the camera. Raw images generally show the surface vasculature of the brain with good contrast (at wavelengths <600 nm), as well as signal originating from deeper layers from light that has been more deeply scattered.

During a stimulus, the intensity of this diffusely reflected light will generally change in a discrete region of the cortex associated with the applied stimulus. This change in detected intensity can be used to map functional regions of the cortex. Early studies identified that a large contributor to changes in diffuse reflectance was haemoglobin absorption [5]. Moreover, haemoglobin is known to have an oxygenation-dependent absorption spectrum in the visible and near infrared range, corresponding to the bright red colour of arterial blood and the darker brown colour of venous blood (figure 2). It was, therefore, recognized that measurements of diffuse-reflectance changes at specific wavelengths across the haemoglobin absorption bands would differently represent contributions from changes in the local concentration of oxy- and deoxyhaemoglobin (Δ[HbO] and Δ[HbR]) as well as their total (Δ[HbT] = Δ[HbO] + Δ[HbR]), which represents a change in local blood volume.

**Figure 3.**
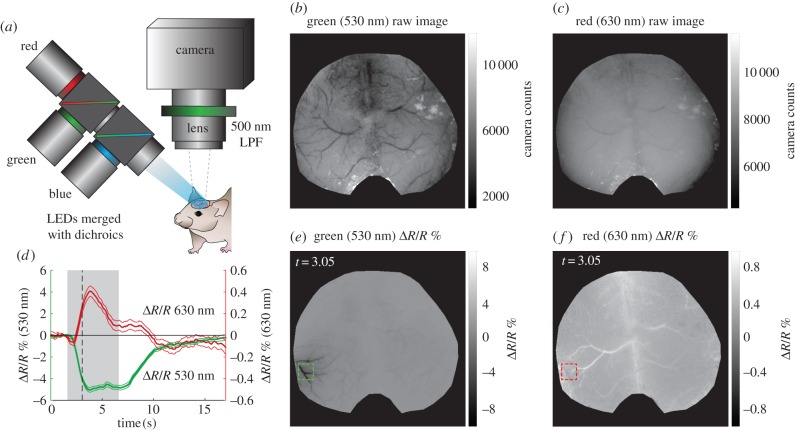
Example of raw reflectance WFOM data in awake mouse. (*a*) Imaging system including blue, green and red LEDs illuminating the surface of the cortex—the resulting reflectance and fluorescence images (500 nm long-pass filter, LPF) are recorded by the camera. (*b*,*c*) Raw, baseline green and red reflectance images of the surface of the cortex. (*e*,*f*) Green and red reflectance changes (%Δ*R*/*R*) at *t* = 3.05 s (1.44 s post-stimulus onset) corresponding to the data shown in figure 1 (response to 5 s, 25 Hz tactile whisker stimulus in awake, behaving mouse, average of 38 repeated trials). (*d*) Temporal profile of Δ*R*/*R* (% reflectance change) in green and red channels during a stimulus from regions indicated in maps to the right. Grey shading shows stimulus time, while black dashed line is at *t* = 3.05 s. Note red changes (right axis) are shown on 10× scale compared to green (left axis).

Figure 3 shows raw reflectance data collected under green (530 nm) and red (630 nm) illumination (corresponding to the converted data shown in figure 1). As described further below, 530 nm is a high-contrast isosbestic point of the haemoglobin absorption spectra meaning that both HbO and HbR have the same absorption values, making the measurement independent of oxygenation and sensitive only to changes in [HbT] (figure 2). The haemodynamic response evoked by stimulation is represented by a darkening of the cortex, corresponding to a local increase in [HbT] (hyperaemia). All vessels appear dark in the absolute image, with high contrast against the background parenchyma. Darker vessel structures in the ratiometic image (below) correspond to dilations. Conversely, 630 nm light is sensitive primarily to [HbR], with a much lower absorption overall compared with 530 nm (figure 2). Veins on the pial surface have the highest baseline concentration of [HbR] as they are the least oxygenated, and thus have darkest contrast in the raw reflectance image at 630 nm. Arterioles have minimal contrast in the raw 630 nm image because they contain almost no HbR. During the later phases of the haemodynamic response, increased blood flow drives an increase in oxygenation, most strongly seen in the veins, reducing the concentration of [HbR] and leading to an increase in reflectance, seen as a whitening of vein structures in the 630 nm ratio image. The ‘initial dip’ period of the 630 nm time course is discussed further in a section below.

### (a) Quantification of haemoglobin changes from diffuse reflectance in the brain

The raw data shown in figure 3 demonstrate that images acquired at different wavelengths carry clear information about the concentrations of HbO and HbR within vessels in the cortex. However, to convert reflectance data into estimates of changes in the concentration of haemoglobin, we must examine the physical principles of light propagation in scattering brain tissue.

In a simple, absorbing but non-scattering medium (such as a glass cuvette filled with a clear, absorbing liquid), the transmission of light will follow Beer's Law:
2.1
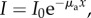
where *I*_0_ is the incident intensity and *I* is the resulting intensity after the light has travelled a pathlength *x* through the medium with absorption coefficient *μ*_a_. However, the brain is highly scattering at visible wavelengths. In fact, scattering is what permits light entering the brain's surface to be diffusely reflected and detected by a camera focused on the brain's surface. As scattering turns the incident light around, each scattering event adds to the photon's pathlength through the absorbing medium. This redirection also introduces spatial uncertainty in terms of where a photon detected at a particular point on the brain originally entered the brain, and where it visited within the brain. Finally, the light emerging from the brain will exit in a range of directions, based on its last scattering event, such that every photon entering will not necessarily be detected by the aperture of the camera lens. In practical implementations, it is impossible to know precisely how scattering has affected each detected photon across the field of view. However, the overall effects of scattering on equation (2.1) can be approximated by the ‘modified Beer Lambert Law’:
2.2
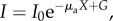
where *X* = DPF*x*, where DPF is the ‘differential pathlength factor’ which represents scaling of the original linear pathlength *x* to account for the longer distance travelled by the photon due to scattering. *G* represents geometric factors that introduce uncertainty regarding how much of the light exiting the brain can be detected by the camera. Calibrating to know exactly the distribution of *I*_0_ on the brain's surface, as well as the geometric factor *G* is challenging to achieve, but it is possible to use relative measurements (e.g. dividing by *I*_(*t*=0)_) to remove the influence of these terms. Incorporating wavelength (*λ*) and time (*t*) dependences, we see
2.3
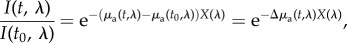
so the change in absorption coefficient from time *t*_0_ to time *t* can be estimated from the ratio of the detected intensity at time *t*, relative to the intensity at time *t*_0_, scaled by a wavelength-dependent estimate of pathlength
2.4
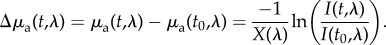
Note that *I*(*t*)/*I*(*t*_0_) − 1 = Δ*R*/*R* shown in figure 3. This analysis assumes that scatter is not changing over time, and that the wavelength-dependent pathlength in the brain is known. These two factors are discussed further below.

Considering the contribution of haemoglobin, assuming that equation (2.4) holds, and if the only change occurring in the brain is the concentration of absorbers in the tissue, the final step in analysis is to use the following relationship:

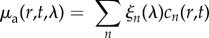
and thus
2.5
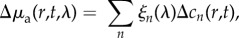
where *ξ_n_*(*λ*) is the molar extinction coefficient of a given chromophore (absorber), Δ*c_n_*(*r*, *t*) is the change in the chromophore's concentration relative to an earlier time point and *r* denotes a two-dimensional pixel location. As shown in figure 2, in the visible range (400–700 nm) the major absorbers in the brain are HbO and HbR. Below 450 nm, protein absorption is higher, while at wavelengths higher than 700 nm, water and lipids can contribute significantly to absorption [27]. However, even if these additional absorbers contribute to baseline absorption, if we consider only changes over time (as in equation (2.4)) the effects of these absorbers will cancel unless their concentrations change over time. In this case, only a slight contribution might be expected from changes in cytochrome oxidase [28]. So using equations (2.4) and (2.5) with a change over time denoted by Δ below, reflectance WFOM measurements at two wavelengths can yield the following pair of equations:
2.6
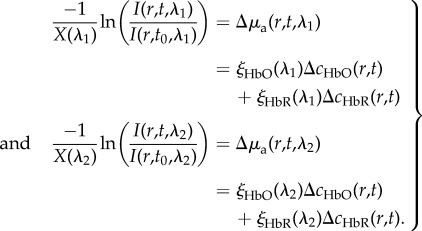
Values of *ξ*(*λ*) correspond to the absolute absorption spectra of HbO and HbR. Values of wavelength-dependent pathlength (*X*(*λ*)) must be estimated. Values of both of these parameters used by our group have been included as a standardization resource in electronic supplementary material, appendix A (haemoglobin spectra reproduced from [26]). With knowledge of *X*(*λ*)) and *ξ*(*λ*), equation (2.6) can be simply solved to find the two unknowns (Δ*c*_HbO_ and Δ*c*_HbR_) at each pixel, which correspond to the time-varying change in molar concentration of HbO and HbR over time
2.7
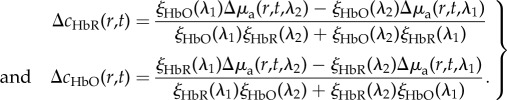
For a larger number of wavelength measurements, this analysis becomes a linear model that can be solved through least-squares fitting or other inversion of the matrix form below. However, the analysis above demonstrates that a minimum of two wavelengths are required to determine (and estimate) or the relative changes in [HbO] and [HbR].
2.8
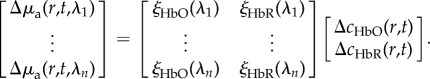


### (b) Scattering governs the spatial resolution and depth sensitivity of reflectance wide-field optical mapping

**Figure 4.**
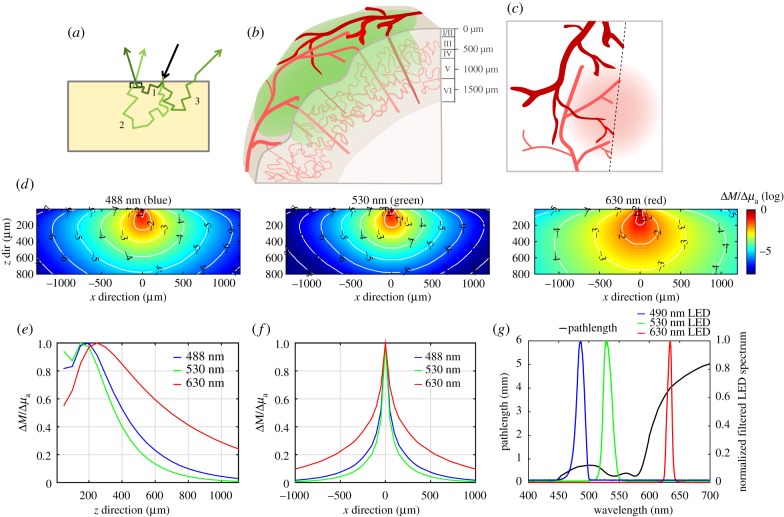
The effects of scattering on spatial sensitivity and pathlength. (*a*) Depiction of light-scattering paths in diffuse reflectance. Photon 1 scatters shallowly and emerges close to the point of illumination. Photon 2 scatters more deeply, becomes less probable but is still detected. Photon 3 scatters and is not emitted within the NA of the detector. (*b*) The vascular architecture of the mouse cortex, where large arteries and veins cover the cortical surface, with diving arterioles feeding deeper capillary beds, which in turn are drained by ascending venules. WFOM illuminates the cortical surface, visualizing surface vessels with sharp contrast. Light penetrating more deeply is attenuated, but can report more blurred signals from capillary contributions as depicted in (*c*). (*d*) Shows Monte Carlo-based simulations of 488, 530 and 630 nm light, showing (*y*-averaged) spatial sensitivity of a camera pixel to changes in absorption at different depths and lateral positions (log_10_ scale on colour bar and contours). Properties: 488 nm: *μ*_a_ = 0.33 mm^−1^, *μ*_s_ = 21 mm^−1^, *g* = 0.8; 530 nm: *μ*_a_ = 0.55 mm^−1^, *μ*_s_ = 21 mm^−1^, *g* = 0.82; 630 nm: *μ*_a_ = 0.024 mm^−1^, *μ*_s_ = 24 mm^−1^, *g* = 0.87, values from [29], and assuming 3% blood in tissue and 75% average oxygen saturation. (*e*) Plot extracted from (*d*) showing the overall depth sensitivity of measurements at a single pixel (over all lateral positions) for the three wavelengths (linear scale) and (*f*) the lateral sensitivity measurements at a single pixel (over all depths). These plots show that red light can detect changes in much deeper tissues than green light (although this difference depends most strongly on absorption differences than scatter). (*g*) Simulated wavelength-dependent pathlength based on Monte Carlo simulations (methods and tabulated values included in the electronic supplementary material, appendix B). Measured spectra of blue, green and red LEDs with band-pass filters are overlaid. Pathlength values (and haemoglobin molar extinction values) used are integrated under these spectral curves for haemodynamic conversion. LEDs used: Thorlabs M490L3 (GCaMP excitation blue), M530L3 (green) and M625L3 (red). Filters used: Semrock FF01-530/43-25 (530/43 in front of the green) and FF01-475/28-25 (475/28 in front of the blue).

The main factors governing the overall depth sensitivity and resolution of reflectance WFOM are the wavelengths of light chosen, the background scattering and absorption properties of the brain and the illumination and detection geometry. As illustrated in figure 4*a*, in a pure, two-dimensional diffuse-reflectance geometry, the most commonly detected photon will be one with a short pathlength, which reversed its direction within a short distance of the surface of the brain and originated from an illumination site close to the detection pixel (figure 4*a* inset, photon 1). Photons that have penetrated deeper into the cortex or travelled further laterally from their site of incidence will have a higher likelihood of being absorbed (equation (2.1)) and will thus contribute less to the overall detected signal (figure 4*a*, photons 2 and 3). This means that signal detected at the cortical surface (in the form of a two-dimensional image) represents a superficially weighted sum of signals from shallow and deeper layers of the cortex as depicted in figure 4*b*.

Scattering properties of tissue are dependent on the wavelength of the light probing the tissue, with the scattering coefficient *μ*_s_ having an approximate spectral dependence of
2.9
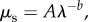
where *b* is estimated to be between 1.3 and 2 for brain tissue [30]. For longer wavelength light (e.g. red compared to blue), both scattering and absorption properties in living tissue are lower (figures 2 and 4). Consequently, red light will, on average, penetrate more deeply into the brain with larger relative contributions to detected signals from deeper changes in absorption compared with blue or green light.

**Figure 5.**
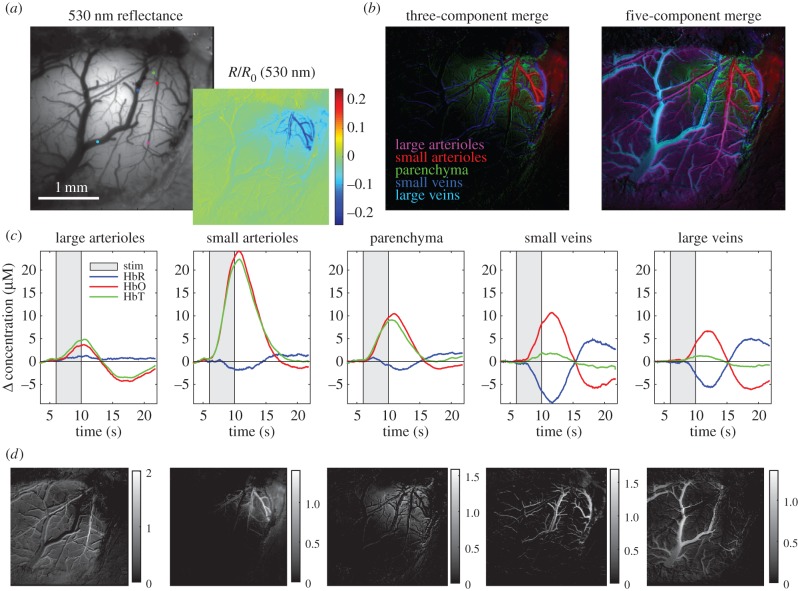
Demonstration of spatio-temporal unmixing of WFOM haemodynamic signals. Data were acquired on an alpha-chloralose anaesthetized rat (from data shown in [11]) during 4 s electrical forepaw stimulation imaged using 470 and 530 nm reflectance measurements, converted to haemoglobin concentrations using equation (2.7). Unmixing method (as described in [31] and in the text) uses basis functions shown in (*c*), sampled from regions in the field of view (indicated by coloured dots in (*a*). Non-negative unmixing yields maps showing the ‘concentration’ of each basis set in the haemoglobin response dataset for each compartment (shown grey scale in (*d*), and as three-component and five-component colour merges in (*b*)). This analysis shows how WFOM can delineate clearly distinct time courses of each vascular compartment despite being a superficially weighted two-dimensional representation of the haemodynamic response.

This spatial sensitivity of the signal detected by a single camera pixel is depicted in results from a Monte Carlo simulation shown in figure 4*d*. The model assumes uniform illumination of the tissue surface with unidirectional light and a low-numerical aperture detection lens (as shown in figure 2*a*). Each map shows *∂*M/*∂μ*_a_(*x*,*z*) on a log_10_ scale, representing the change in measurement M that would result from a spatial change in absorption *μ*_a_ at each location (*x*,*z*), based on estimates of the optical properties of brain for each wavelength (see caption for assumptions, see electronic supplementary material, appendix B for full details of model). Figure 4*d,e* show laterally averaged and depth-averaged contributions, respectively, for each wavelength. These plots show (for the simulation parameters chosen) that changes in absorption at depths up to and exceeding 500 µm can contribute to signals detected in reflectance WFOM, although photons probing deeper changes contribute less towards the detected signal than photons probing more superficial tissue. This spatial sensitivity implies that surface vessels will contribute more strongly to changes in signal, but that changes in deeper capillaries (even if blurred) should be detectable in systems with sufficient dynamic range and signal to noise as depicted in figure 5. The higher sensitivity to tissues just below the surface (rather than the very surface) corresponds to the need for the photons to reverse their direction in order to be detected.

Despite the assumptions implicit in spectral conversion and in integrated two-dimensional measurements, the sensitivity of reflectance WFOM data to different vascular compartments can be confirmed using spatio-temporal unmixing as shown in figure 5. This approach recognizes that there is an expected difference in the haemoglobin dynamics of each vascular compartment, for example, arterioles will have high HbO concentrations and will increase in diameter (increasing [HbT]) but with little change in [HbR]. Veins are expected to show strong decreases in [HbR] as fresh, oxygenated blood washes into the system during hyperaemia, with relatively little dilation and thus minimal changes in [HbT]. As described further in [31], unmixing proceeds by selecting a small region of each identified vascular compartment within the responding region (after averaging WFOM signals over multiple stimulus repetitions). [HbO], [HbR] and [HbT] changes are then extracted from these ‘seed regions’ and concatenated across time, forming a basis set of vascular signatures (*B*_Comp, Hb___All_(*t*)). Non-negative least-squares fitting at every pixel is then performed on concatenated changes in haemoglobin concentrations (Δ*c*_Hb___All_(*r*,*t*)) to determine the contribution of each time course to the signal in each pixel (assuming a linear sum as shown in equation (2.10)). The results are individual maps *P*_Comp_(*r*) delineating the presence of each dynamic signature in each pixel. Maps that generate low residuals and expected vascular patterns confirm good representation of the compartment time course in the compartment-specific basis time courses.
2.10
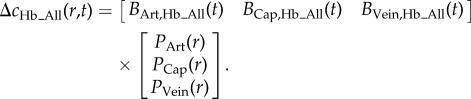


Figure 5 shows unmixing of five temporal haemodynamic components ranging from larger arteries to smaller arterioles, capillaries, venules and veins. Image merges of colour-coded spatially resolved compartments are shown for three- and five-compartment fits along with basis time courses. This analysis clearly delineates capillary contributions that have a different (and expected) time course to arteries or veins, but are more blurred, appearing as a background to the image compared to the sharp relief of the surface vessels (cf. figures 1 and 3).

### (c) Limitations of using an estimated, average, wavelength-dependent pathlength

The analysis shown above confirms the utility of WFOM for spatio-temporal haemodynamic analysis. However, it should be remembered that spectroscopic conversion of WFOM data relies upon several major assumptions that require careful consideration before small nuances in response maps and timing are assumed to be accurate. In particular, the modified Beer Lambert Law (equation (2.2)) requires a pathlength estimate to account for the distance travelled by light in the brain due to scatter. It is important to understand the effects of this pathlength assumption on the accuracy of WFOM spectral calculations:

(1) The pathlength *X* used represents an average value for all of the light detected from the cortical surface. Each pixel is detecting a multitude of photons that have travelled a range of different distances within the tissue (figure 4*a*). The modified Beer Lambert Law must thus be valid for the detected distribution of photons and their pathlengths.
(2) Light of different wavelengths will, on average, probe different volumes of the brain, and particularly different depths (as shown in figure 4*d*–*f*). This wavelength-dependence is potentially problematic because equation (2.5) and thus equation (2.7) rely upon measurements that describe a unique mixture of chromophores. Thus, if there is significant heterogeneity of changes in absorption, measurements at different wavelengths could yield data that are inconsistent with this linear model resulting in conversion errors. It is, therefore, advantageous, where possible, to use wavelengths with comparable scattering properties for haemodynamic imaging (factors influencing wavelength choice are detailed below).
(3) Estimation of pathlengths for spectroscopic conversion requires an estimate of the scattering and absorbing properties of the brain, as well as knowledge of the illumination and detection geometry. As shown in equation (2.9), scattering in brain decreases with increasing wavelength. However, this dependence is not the only source of wavelength-dependence for pathlength estimation. The absorption properties of the tissue are also wavelength-dependent (e.g. corresponding to the spectrum of haemoglobin). The effect of this wavelength-dependence of absorption on estimation of pathlength can be understood by considering that high absorption will lead to stronger attenuation of photons that have travelled furthest (by equation (2.1)), which will decrease the average pathlength of detected photons at that wavelength. Absorption is a stronger factor affecting average pathlength than scattering.

The commonest approach to estimating pathlength is to use values for *X* calculated using a Monte Carlo model of light propagation based on estimates of the brain's background scattering and absorption properties [32,33] (see electronic supplementary material, appendix B for Monte Carlo model details and example values for *X*). In general, we assume that the absorption spectrum of the brain can be estimated using equation (2.6), assuming 2 mM concentration of haemoglobin in blood, 3% blood in tissue by volume, and an average haemoglobin oxygenation of 75% [30] (see electronic supplementary material, appendix A for haemoglobin absorption spectra and conversions). It is possible to go beyond this simple modelling and incorporate spatial heterogeneity into estimates of the baseline absorption and scatter properties of the brain, or even a nonlinear analysis that re-computes pathlength based on inferred changes in absorption [8,34,35]. However, this is both computationally intensive and introduces the risk of erroneous divergence of the algorithm.

In general, published values for optical pathlengths in the rodent brain have varied by a factor of 3, ranging from low values of 0.3–1 mm at 540–580 nm to 3–7 mm around 680 nm [8,36]. The use of these different factors between groups means that inferred values for Δ[HbO], Δ[HbR] and Δ[HbT], often shown in terms of absolute micrometre concentrations, are somewhat subjective. Ironically, the logarithmic nature of Beer's Law means that values of Δ*R*/*R* (fractional change in diffuse reflectance = Δ*I*(*t*)/*I*(*t*_0_) from equation (2.3) for specific wavelengths *are* robust measurements that should be repeatable and comparable between groups thanks to the cancellation of experimental variables such as the illumination intensity distribution. It would, therefore, be valuable to report both raw Δ*R*(*λ*)/*R*(*λ*) values for responses (as shown in figure 3) in addition to spectrally converted values for Δ[HbO], Δ[HbR] and Δ[HbT], in cases where the relative amplitude of haemodynamics is a valuable parameter to compare between groups. Alternatively, the use of common pathlength values could also assist in this standardization. Our routinely used pathlength values are plotted in figure 4*g* (and tabulated in electronic supplementary material, appendix B for this purpose), noting however, that these values should be carefully validated for different experimental conditions, animal models and experimental paradigms.

### (d) Choice of wavelengths

Several factors affect the choice of suitable wavelengths for multi-spectral reflectance WFOM. First, it is often helpful to choose at least one wavelength that is isosbestic (*λ*_iso_), such as 530 nm as described above. In this case, reflectance measurements obtained can be assumed to be invariant with changes in haemoglobin oxygenation. Although this reduces information, this measurement can be reliably considered as a measure of changes in blood volume within the tissue, independent of pathlength estimates or values for haemoglobin absorption coefficients. From equations (2.5) and (2.6)
2.11
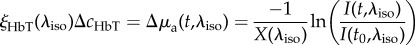
and so irrespective of pathlength estimates or haemoglobin spectra
2.12
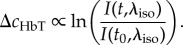
It should be noted, however, that unless a very narrow wavelength range is used, the spectral bandwidth of light centred on an isosbestic point could introduce some sensitivity to oxygenation.

Choice of additional wavelengths should consider several additional factors: (i) that the wavelength has good sensitivity to oxygenation-dependent changes, ideally selecting two spectral regions where HbO and HbR absorption dominate, respectively (figure 2), to give counteracting measurements. From equation (2.3), it can also be seen that the percentage changes in signal (Δ*I*/*I*) for a given change in [HbT] will be higher for wavelengths around blue and green, where the absorption coefficient of haemoglobin is much stronger, than in the red, although note that shorter wavelengths also yield less depth penetration than longer wavelengths (figure 4). (ii) The baseline scattering and absorption experienced by the wavelengths are not excessively different (as explained above), noting that longer red wavelengths will penetrate around two times more deeply into the brain than blue and green light (figure 4*d*–*f*). (iii) Availability of suitable sources of illumination with sufficient power in sufficiently narrow spectral bands to allow accurate estimation of haemoglobin absorption parameters (this is much easier since the advent of high power light emitting diodes (LEDs) at a wide range of wavelengths). It should be noted that lasers are less often used as light sources owing to the problem of speckles in wide-field images. (iv) Ensuring avoidance (or in some cases deliberate overlap) of spectral bands important for other aspects of the experiment such as GCaMP excitation and emission ranges, absorption bands of channelrhodopsins [37] and absorption bands of agents such as photosensitizers used to cause photothrombic strokes.

A typical set of wavelengths used in our experiments is 490 nm (blue, more sensitive to HbO and corresponding to GCaMP excitation), 530 nm (green, an isosbestic point) and 630 nm (red, more sensitive to HbR) (as shown in figures 1 and 3) [11,13,19,24]. Illumination is provided by filtered, strobed LEDs as depicted in figure 3*a*. Although combining 490, 530 and 630 nm signals may introduce some spatial sampling confounds as described above, all conversions are tested in the following way: given three wavelengths (e.g. A, B and C), conversions to Δ[HbO] and Δ[HbR] are performed using successive pairs of wavelengths via equation (2.7) (e.g. A&B, B&C and A&C). If all three conversions yield traces of Δ[HbO] and Δ[HbR] that are in agreement, conversion assumptions are assumed to hold [38].

### (e) Does scatter change during functional activity?

The analysis above does not consider scattering to be a time-varying function during functional changes. Changes in the scattering properties of tissue can affect signal detected in a diffuse-reflectance geometry in counteracting ways: (i) increases in scatter will increase the pathlength of light within the absorbing medium, which should decrease detected light intensity. (ii) Conversely, increases in scatter could improve redirection of light and thus increase the number of photons rapidly reversing their direction and exiting the brain to be detected by the camera. (iii) Increased scatter will cause photons travelling deeper into the tissue to be more heavily absorbed, such that detected signal will become more weighted to superficial layers. All of these effects in combination can lead to ambiguous interpretations of increases and decreases in intensity being attributable to increases or decreases in scatter [39]. Thus, although it is often assumed that a change in scatter can be modelled as another absorbing term in equation (2.1), the effects of scattering can be complex in a diffuse-reflectance geometry.

Foundational work performed in bloodless *in vitro* tissues such as the giant squid axon detected small (approx. 10^−6^) changes in the intensity of directionally scattered light that can be clearly attributed to small changes in scattering [40]. However, very few reports in the living, mammalian brain have provided clear evidence of a detectable signal change caused by scatter changes. One paper that shows compelling evidence for a small, very fast scattering signal is Rector *et al.* [6]. The authors used 660 nm light (red) to avoid strong haemoglobin absorption, and a dark-field illumination geometry to maximize penetration depth and reduce opposing, back-scatter enhancing effects of increases in scatter. In response to a single whisker stimulation, the authors observed a small (Δ*I*/*I* ∼ −0.01%), very rapid decrease in detected optical signal that reached its minimum within 200 ms of stimulus delivery and corresponded well to electrical recordings. This signal was found to map accurately to the individual barrels of the rat whisker-barrel region of the cortex. These measurements are likely accurate representations of changes in brain scattering, but it should be noted that such changes due to neural activity are very fast, and highly challenging to detect.

Although early intrinsic signal imaging studies proposed that slower changes in cortical reflectance could, in part, be due to neural activity-linked changes in brain scattering, this possibility is not consistent with the observations of Rector *et al.* [5]. It has also been suggested that changes in blood flow could alter the scattering properties of the brain [41], although this effect has not been quantified *in vivo*. It is concluded that neurally correlated changes in brain scattering are likely to be only very minor, fast contributors to signals detected in reflectance WFOM measurements. It is thus assumed here that changes in scattering do not need to be incorporated into standard analysis to extract haemoglobin concentrations from multi-spectral WFOM data.

### (f) Representations of the ‘initial dip’

The first description of the ‘initial dip’ was presented in an early study by Malonek & Grinvald [5] who acquired cortical reflectance data using a slit spectrometer. This study demonstrated the presence of the characteristic spectral bands of haemoglobin absorption (figure 2), indicating a haemodynamic origin for ‘intrinsic signals’. After spectral conversion, the Δ[HbR] trace was found to show a well-localized early increase in [HbR], interpreted as initial consumption of oxygen (an ‘initial dip’), followed by an undershoot in [HbR] corresponding to an increase in oxygenation as fresh blood washes into the active region. This result was exciting as it indicated that the early component of the ‘intrinsic signal’ could represent a direct measure of neuronal activity in the form of oxygen consumption.

Following this result, it was noted that reflectance WFOM data acquired at a single wavelength between 600 and 640 nm would almost always yield a signal in response to stimulus that initially dipped, and then overshot similarly to the inverse of the Δ[HbR] trace shown in [5,42] (this trend can be seen in the 630 nm reflectance data shown here in figure 3). It was reasoned that HbR is the dominant absorber in this range, and therefore, the signal was an accurate representation of changes in [HbR] (figure 2) without any further need for spectral conversion. As a result, many researchers interested in mapping cortical regions (e.g. to guide electrophysiology) used only a single red wavelength for ‘intrinsic signal imaging’ and focused only the early part of the response under the assumption that it equates to neural oxygen consumption [42–44].

However, several subsequent studies questioned the original ‘initial dip’ result, showing that adequately accounting for the wavelength-dependence of pathlength during spectral conversion results in an [HbR] trace that exhibits no initial increase [14,45,46]. The presence of the initial dip has also been challenged by many in the functional MRI community, where only a few groups report seeing an equivalent change in the blood oxygen level-dependent signal (which should be inversely proportional to [HbR], such that an ‘initial dip’ would correspond to deoxygenation [47]). Spectral analysis in Malonek & Grinvald [5] both did not account for wavelength-dependent pathlengths and was also performed under the assumption that large changes in scattering were occurring, and that scatter changes would add linearly to a model of the form shown in equation (2.6). This model fit predicted light-scattering changes that first decreased and then increased in a pattern that closely resembled the inverse of the trace derived for Δ[HbR]. Had scattering been held constant, the model might have yielded an Δ[HbR] trace with a monotonic decrease. Moreover, the derived Δ[HbO] trace exhibits a smooth increase immediately following stimulus onset, consistent with a rapid increase in local perfusion that should counteract local oxygen consumption.

Although some groups performing spectral conversion of multi-spectral WFOM data do report initial dips (initial [HbR] increases) [48,49], many other studies performing spectral conversion show [HbR] traces that exhibit no initial dips [50–52]. In our case, despite being present in our raw red reflectance time course in figure 3, an initial dip in [HbR] is not seen in spectrally converted data shown after conversion in figure 1.

In an attempt to explain and reconcile these results, we performed a reflectance WFOM study in the awake, behaving primate visual cortex using green 530 nm (isosbestic) and two red, 605 and 630 nm recordings [53]. Earliest changes in reflectance were seen in 530 nm signals, which by equation (2.12) should accurately report changes in total haemoglobin [HbT] concentration. However, simultaneously acquired changes in 605 and 630 nm signal showed a characteristic dip in signal, followed by an overshoot (consistent with figure 3). After spectroscopic conversion, this dynamic pattern of red light reflectance was demonstrated to be attributable to the small, but finite 10–20% contribution of HbO absorption to reflectance in this red wavelength range (highlighted in figure 2). A large initial increase in [HbT] (as indicated by a decrease in 530 nm green reflectance), which would be mostly driven by increases in [HbO], since arterial blood is entering the area, would cause a signal decrease at 600–650 nm owing to a higher overall concentration of absorbers. Subsequent decreases in the concentration of [HbR] from this inflow would then dominate the signal and lead to an overshoot in red reflectance. Conversion of these data using a broad range of pathlength factors all resulted in calculated time courses for [HbR] that exhibited monotonic decreases, without an initial increase in [HbR], despite raw red reflectance data having what appeared to be an initial dip (consistent with our converted data shown in figure 1) [53].

This result does not question whether ‘initial dips’ (initial deoxygenations) can occur in the brain: it is highly likely that certain brain areas [36,54], anaesthesia states, disease states, developmental stages [19] or types of task could yield an imbalance between oxygen consumption and the arrival of fresh blood. It could also be argued that errors in spectral conversion of reflectance WFOM data, or effects of initial scattering changes, could be erroneously eliminating an initial increase in calculated [HbR] [55]. There is also clear evidence that early oxygen consumption takes place, and initial decreases in tissue PO_2_ following stimulation have been convincingly measured [45,55,56]. However, the results described above show that the standard use of a single 600–650 nm red light source for ‘intrinsic signal imaging’ can be ambiguous and misleading. Interpretation of the biphasic shape of red reflectance as an initial dip (corresponding to oxygen consumption) followed by an overshoot (corresponding to perfusion) does not take into account the small, yet significant absorption of HbO at these wavelengths. Initial decreases in red reflectance thus more likely correspond to early, localized hyperaemia in the capillary beds [57,58] that are also evident at isosbestic green (530 nm) wavelengths, rather than being faithful representations of oxygen consumption.

## 3. Wide-field optical mapping of fluorescence contrast

In addition to haemodynamic and ‘intrinsic signal’ optical imaging, the exposed brain can also be imaged to capture a range of dynamic changes in fluorescence contrast including voltage [1,59] and calcium sensitive dyes [24,60,61], genetically encoded calcium [62–65] and voltage indicators [66–68] and intrinsic fluorophores as markers of oxidative metabolism [18,19,69–74].

Instrumentation for imaging fluorescence contrast in a wide-field geometry can be almost identical to that used for reflectance WFOM. The major practical difference is that fluorophores excite with lower wavelength (higher energy) light, and emit at higher wavelengths (lower energy light). This means that the brain must be illuminated at the fluorophore's excitation wavelength, and this wavelength must be blocked from entering the camera to enable sensitivity to photons at the fluorophore's emission wavelengths. Emission intensities are typically much lower than excitation energies. It is argued here that reflectance WFOM measurements should always be acquired in parallel with fluorescence measurements to protect against haemodynamic cross-talk as explained further below.

Overall, the imaging properties of WFOM for fluorescence imaging are largely similar to those of haemodynamic imaging. However, there are several considerations for fluorescence WFOM that differ from reflectance imaging as described below:

### (a) Depth sensitivity of fluorescence wide-field optical mapping

**Figure 6.**
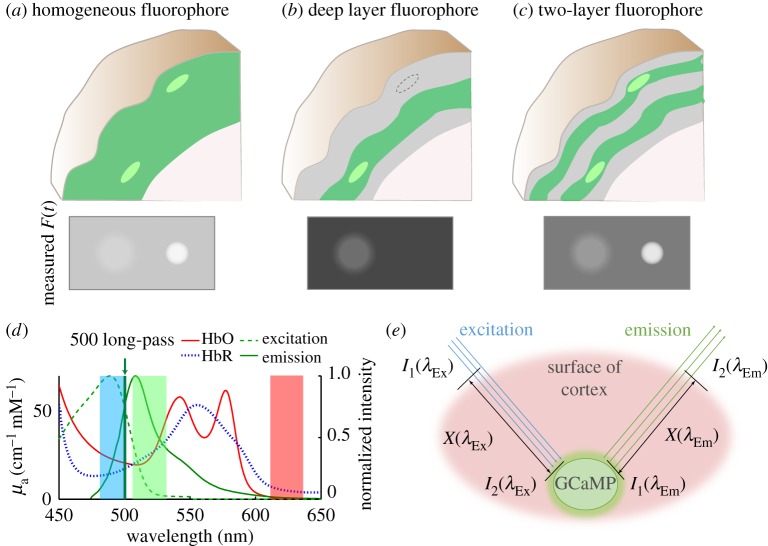
Spatial dependence of fluorescence WFOM sensitivity and haemodynamic cross-talk. (*a*–*c*) Depict different patterns of fluorescence expression that could be generated in the brain (indicated by green shading). Brighter regions are intended to denote a region of increased intracellular calcium. Panels below show estimated images of fluorescence that could be detected at the cortical surface in each case. (*d*) The absorption spectra of HbO and HbR overlaid with the excitation and emission bands of GCaMP, as well as bands indicating LED wavelengths used for WFOM results shown herein. (*e*) Schematic of light paths in WFOM travelling to and from a GCaMP fluorescence interaction.

An important distinction between reflectance and fluorescence WFOM relates to the effects of fluorophore spatial heterogeneity on the spatial dependence of imaging. As illustrated in figure 6*a*–*c*, if a fluorophore is located in a layer deep within the brain (figure 6*b*), and fluorescent light is detected by the imaging system, the detected signal must have originated from the fluorescent layer [75]. This is different from the case of diffuse reflectance (as demonstrated in figure 4) where the superficial layers will always be the regions of highest sensitivity to absorption changes (unless dark-field illumination strategies are employed [6]). This effect brings the benefit that light (albeit attenuated and spatially blurred from scattering) could be selectively detected from fluorophores located deep within the brain if labelling was restricted to only this deep region, sources were bright enough and detectors had sufficient sensitivity. However, if more superficial layers are also fluorescent (figure 6*c*), sensitivity will be much more strongly weighted to fluorescent regions closet to the surface, increasing background as well as the dynamic range required to also detect changes in deeper regions. The other effect of this difference is in the applicability of using diffuse-reflectance measurements to correct for absorption contamination of fluorescence measurements as described in more detail below.

Another slight difference between the spatial sensitivity of wide-field fluorescence and absorption measurements comes from the fact that fluorescence emissions are isotropic. Diffuse-reflectance signals come from photons that have repeatedly scattered, usually in a fairly forward direction, until they emerge from the tissue (with signal being inferred from photons that are absorbed, and thus do not emerge). Conversely, a fluorescent absorption event can occur for a photon travelling in any direction, and emission of a fluorescence photon will be in a random direction, such that photons with effectively shorter paths could feasibly return to the detector more efficiently than in diffuse reflectance [76]. This effect is accounted for in the Monte Carlo simulations of depth sensitivity shown in figure 4 and described further in electronic supplementary material, appendix B.

Finally, for one-photon interactions, it should be noted that excitation light will always be of a different (lower) wavelength than emission light, and that these two wavelengths will experience different absorption and scattering properties within the tissue (figures 2 and 4). Even if a fluorophore emits at longer wavelengths where light propagation will be improved, if blue (for example) light is required for excitation, the penetration depth of this excitation light will remain a limiting factor. Correction for fluorescence contamination by absorption of haemoglobin must also account for attenuation of both excitation and emission bands, as described further below.

### (b) Image resolution limitations for fluorescence wide-field optical mapping

The type of fluorophore being imaged will dictate which physical structures are labelled. In the case of the calcium-sensitive fluorophore GCaMP, specific cell types can be selectively targeted to express the fluorescent protein, from all neurons in the cortex, to specific sub-types or layers, dendrites or cell bodies, sparsely or densely labelled, or even other reactive cells such as astrocytes [62–65]. Other labelling strategies can yield more diffuse labelling such as VSDs which localize to cell membranes, or FAD which is located in mitochondria [1,59,77].

In general, the ability of fluorescence WFOM to spatially resolve single-cell activity can be considered equivalent to the challenge of using a low numerical aperture epi-fluorescence microscope to image a large intact sample. If cell bodies are located close to the surface, where light will have undergone very few scattering events, and sufficient magnification is used, individual cells can likely be resolved but will have low contrast owing to out-of-plane background signal. In the intact brain, very superficial structures such as apical dendrites in layer 1 can be discerned to flash if sparsely expressing GCaMP. An emerging way to establish close proximity to cell bodies deeper within the cortex is to implant a gradient index (GRIN) lens into the brain and perform WFOM over the relatively small two-dimensional field of view at the GRIN lens's tip [78]. Resolving single cells or structures can be enhanced by extracting changes in fluorescence corresponding to temporal events such as neuronal firing, and extended via the use of spatio-temporal unmixing strategies to extract signals from additional cells beyond the focal plane [79] similar to the haemodynamic unmixing strategy shown in figure 5 [31,80].

However, for measurements wishing to probe changes in fluorescence in larger volumes, beyond the initial few scattering events of light, blurring will reduce the ability to resolve and separate activity in individual cells. In the data shown in figure 1, each camera pixel captures an area of around 20 × 20 µm. The purpose here was to capture neural activity across the entire superficial cortex of the mouse (including multiple cortical depths) in parallel, rather than seeking single-cell resolution. Here, the combined scattering effects and temporal properties of GCaMP provide a spatio-temporally integrated ensemble representation of neural activity in each region.

### (c) Background subtraction for ratiometric calculations

Fluorescence measurements are typically reported as Δ*F*/*F*, the fractional change in fluorescence relative to some baseline, e.g. at time *t* = 0. The true fluorescence of the fluorophore *F*_True_(*r*,*t*) is affected by the following systematic effects: (i) illumination uniformity, detection uniformity and the spatial uniformity of fluorophore expression (collectively represented by *S*(*r*) below, where *r* denotes position). (ii) Time-varying confounds such as haemodynamic absorption, given by *P*(*r*,*t*) and (iii) ‘dark’ signal *D*(*r*) given by the background signal measured when taking an image with identical acquisition settings, but without illumination of the brain, and will capture both extraneous ambient light and the camera's baseline ‘dark level’ which can be up to 100 counts. The measured fluorescence *F*_Meas_ is then given by
3.1
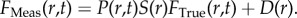


Ratiometric analysis is intended to cancel out these systematic errors (as it does for reflectance WFOM). For fluorescence, however, as in equation (3.2) compared with equation (3.3), it is essential to first subtract the dark signal *D*(*r*), to enable *S*(*r*) to cancel out. This analysis is true for all WFOM analysis, but is particularly important for fluorescence measurements where detected counts on the camera can be small in comparison with the amplitude of *D*(*r*)
3.2
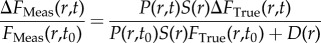

3.3
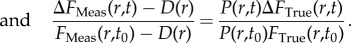


Even after ratiometric analysis it is clear that fractional changes in fluorescence will not be corrected for time-varying confounds such as absorption changes, as described further below.

### (d) The need for haemodynamic correction of wide-field fluorescence data

Figure 6*e* illustrates the simplified paths taken by light to and from an interaction with a fluorophore. Excitation light must travel a pathlength *X*(*λ*_ex_) through the brain before reaching the fluorophore, and along this path will experience the absorption properties of the tissue it travels through. An equivalent effect will attenuate light emitted from the fluorophore. As shown in figure 6*d*, the excitation and emission bands of GCaMP overlap with strong (and different) absorption bands of HbO and HbR. If background absorption properties remained constant, these effects would have minimal consequences; however, through the process of neurovascular coupling, almost all neural activity should be accompanied by functional hyperaemia [81]. As evidenced by the strongly detectable signals in reflectance WFOM, these changes in haemodynamics will thus modulate detected fluorescence, contaminating fluorescence recordings. From figure 6*e*, we can formulate a simple model of this effect:
3.4
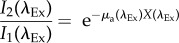

3.5
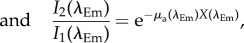
where *I*_1_(*λ*_Ex_) represents the initial intensity of excitation light entering the tissue, *I*_2_(*λ*_Ex_) represents the intensity of the excitation light reaching the fluorophore after travelling pathlength *X*(*λ*_Ex_) through tissue with absorption coefficient *μ*_a_(*λ*_Ex_). Similarly *I*_1_(*λ*_Em_) represents the initial intensity of fluorescent emission light and *I*_2_(*λ*_Ex_) is the intensity of emission light exciting the tissue after travelling pathlength *X*(*λ*_Em_) through tissue with absorption coefficient *μ*_a_(*λ*_Em_). Conversion of excitation light to fluorescence by a fluorophore is a function of *σ*, the fluorophore's conversion efficiency and *c*_f_, representing either the concentration of the fluorophore or the effect (e.g. concentration of calcium) that fluorescence is proportional to. Therefore,
3.6
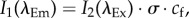
By substituting equations (3.4) and (3.5) into equation (3.6), the intensity of fluorescence emission light emerging from the tissue is given by
3.7
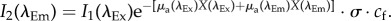
As shown above in equation (3.3), the ratio of measured fluorescence relative to a baseline state *F*_Meas_(*t*_0_) (after dark subtraction) should cancel out multiplicative, time-invariant factors *S*(*r*) such as the unknown distribution of excitation light *I*_1_(*λ*_Ex_), as well as the fluorescence conversion efficiency *σ*, leaving
3.8
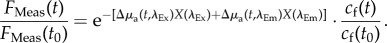


The above equation is a very important result as it demonstrates that the effects of absorption (haemodynamic) cross-talk are multiplicative with respect to the ratiometric change in intracellular calcium. This relationship means that using brighter GCaMPs or other fluorophores will not overcome the effects of haemodynamic cross-talk owing to their larger signal amplitude.

The result does, however, highlight the dependence of cross-talk on the pathlength travelled by photons on their way to and from the fluorophore. Returning to figure 6*a*–*c*, the spatial distribution of fluorophores can thus affect the magnitude of haemodynamic cross-talk affecting data. In the case of a brain expressing strong, uniform fluorescence, most fluorescent light will be detected from superficial layers, photons that will have travelled a shorter pathlength and will, therefore, carry exponentially less haemodynamic contamination than in measurements of fluorophores situated in deeper layers.

### (e) Methods of correcting for haemodynamic cross-talk in fluorescence wide-field optical mapping

To correct fluorescence WFOM data we must remove the time-varying absorption term from equation (3.8) (*P*(*r*,*t*) in equation (3.3)). We note from equation (2.3) that diffuse-reflectance measurements give us an approximation to this absorption term, if measured at the excitation or emission wavelengths of the fluorophore. In the case of fairly broadband 530 nm (green) diffuse reflectance, which approximates the emission band of GCaMP:
3.9
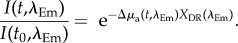


The main difference is the pathlength term *X*_DR_(*λ*_em_), with DR denoting diffuse reflectance, corresponding to light entering the tissue, scattering and emerging. This distance is not the same as the pathlength term *X*(*λ*_em_) in equation (3.8) which corresponds to the light's path from the fluorophore to the tissue surface. Nevertheless, this formulation demonstrates that simultaneously acquiring green reflectance data (or the fluorophore's emission band) while acquiring fluorescence WFOM data will provide a valuable measure of the spatio-temporal properties of absorption changes that could confound interpretation of fluorescence changes.

Mathematically removing contributions from changes in absorption can be achieved, but will again each require approximations. We have developed three different methods for correction as detailed below:

(1) Approximate division/regression of single-wavelength reflectance data.
(2) Removal of excitation and emission contamination using multi-spectral reflectance.
(3) Blind-source removal of haemodynamic contamination.

#### (i) Haemodynamic correction using only green diffuse reflectance (single-wavelength method)

The first approach to haemodynamic correction is to use simultaneously recorded 530 nm (green) diffuse-reflectance signals. In this case, the properties of the tissue (and changes in absorption) are assumed to be the same at *λ*_ex_ and *λ*_em_ (so Δ*μ*_a_(*t*,*λ*_em_) ≈ Δ*μ*_a_(*t*,*λ*_ex_)) and the pathlength of the 530 nm diffuse reflectance light is assumed to be approximately the sum of the excitation and emission pathlengths (*X*_DR_(*λ*_em_) ≈ (*X*(*λ*_ex_) + *X*(*λ*_em_)). Contamination removal (under these approximations) can then be done by simply dividing the fluorescence ratio by the reflectance ratio (from equations (3.8) and (3.9))
3.10
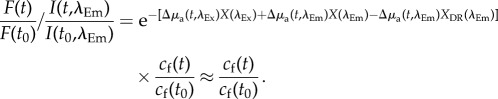


#### (ii) Haemodynamic correction using estimated excitation and emission attenuation (Ex–Em method)

A more rigorous way to correct the haemodynamic confound is to correct for attenuation changes at both the excitation and emission bands of the fluorophore. In practice, acquiring reflectance images at the fluorophore's excitation band is difficult to achieve for high frame-rate imaging using a single camera, since a long-pass filter would need to be modulated in front of the camera, or a second camera would be needed. Instead, we show here that an improved correction can be achieved via dual-wavelength reflectance measurements that do not necessarily include the fluorophore's excitation band.

In the case of figure 1, GCaMP is excited at 488 nm and diffuse reflectance is measured at 530 and 630 nm. Based on the theoretical framework presented above, spectroscopic analysis (equation (2.7)) should be able to infer the corresponding time-varying changes in [HbO] and [HbR] for each pixel using these 530 and 630 nm reflectance measurements. Equation (2.6) can then be used to calculate the effective cortical Δ*µ*_a_ at the 488 nm excitation wavelength (or any other wavelength) at the same point in time. We can, therefore, formulate an absorption part of the correction factor as:
3.11
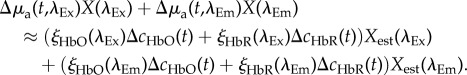


Although pathlength values *X*_est_ in this case must be estimated, this additional degree of freedom can help in accounting for the inherent differences in *X*_DR_(*λ*) and *X*(*λ*), enabling a better approximation to the fluorescence case, and differences in absorption and pathlength properties at excitation and emission wavelengths. In practice, values of *X*_est_(*λ*_Ex_) = 0.56 mm^−1^ and *X*_est_(*λ*_Em_) = 0.57 mm^−1^ in Thy1-GCaMP mice have been found to yield corrected GCaMP images with minimal vessel artefacts and low-frequency trends resembling haemodynamic cross-talk.

**Figure 7.**
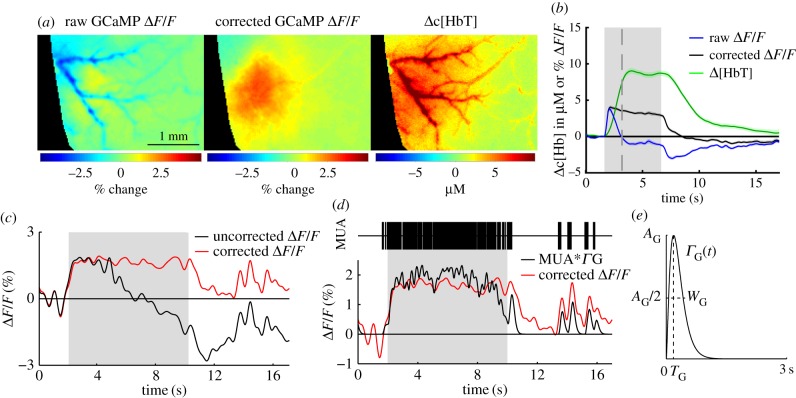
Haemodynamic correction of WFOM GCaMP data and validation with electrophysiology. (*a*) Data shown in figure 1 before and after haemodynamic correction using the Ex–Em method. Maps are for 1.7 s after onset of a 5 s tactile whisker stimulus in an awake, behaving mouse (average of 38 trials). Corrected image shows only minor vessel artefacts, whereas original response is dominated by negative contrast corresponding to the haemodynamic response. (*b*) Time courses show the GCaMP signal before and after correction, in comparison with the D[HbT] haemodynamic response. (*c*) Raw uncorrected and single-wavelength ratiometrically corrected and GCaMP signals acquired in a urethane-anaesthetized Thy1-GCaMP3 mouse undergoing an 8 s electrical hindpaw stimulation. (*d*) An electrode inserted into the responding region recorded multi-unit activity (MUA). The GCaMP fluorescence signal after haemodynamic correction can be closely replicated by convolving MUA with a gamma function (*e*) to model the combined dynamics of intracellular calcium and GCaMP fluorescence changes.

The Thy1-GCaMP6f images and time courses shown in figure 1 were corrected using the Ex–Em method and pathlength values described above. Figure 7 shows an inset of the same data before and after haemodynamic correction. The GCaMP image before correction shows strong vessel artefacts corresponding to absorption by haemoglobin, which are removed after correction. The time course of the uncorrected GCaMP trace for a 5 s tactile whisker stimulation, peaks at first and then dips below baseline as the haemodynamic response reaches its peak. After correction, the GCaMP signal stays positive for the duration of the stimulus. To verify this correction, figure 7 also shows a comparison between wide-field GCaMP measurements and electrophysiological recordings at the same site. Data were acquired in a urethane-anaesthetized Thy 1-GCaMP3 animal during an 8 s duration, 3 Hz electrical hindpaw stimulation. Electrophysiological data were spike-sorted to yield multi-unit activity (MUA). Raw fluorescence data were corrected using the single-wavelength method. A convolution of MUA with a best-fit gamma function representing the kinetics of GCaMP yielded a good fit to haemodynamic-corrected WFOM fluorescence data. This result both confirms that haemodynamic-corrected data are a more precise representation of neural data than raw fluorescence, while also demonstrating that WFOM fluorescence in Thy1-GCaMP mice provides a valuable representation of underlying excitatory activity [19].

#### (iii) Haemodynamic correction by blind-source separation

In cases where diffuse-reflectance measurements cannot be (or have not been) acquired, it may be possible to extract the haemodynamic component using blind-source separation techniques such as principal component analysis (PCA) [19]. Such methods assume that data can be modelled as a linear sum of temporal components, and seek the largest contributors to variance within the data (which here is multiple time courses given by all of the pixels in the image). The most important consideration in this approach is to note that haemodynamic contamination of fluorescence data is *multiplicative* (equation (3.8)), whereas PCA and associated methods assume that data are composed of a *linear* sum of separable components. As such, PCA for contamination removal should be done on the logarithm of fluorescence data, which reduces the contamination problem to a linearly added component (from equation (3.8))
3.12
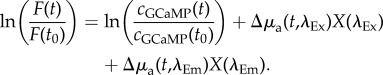


In some cases, PCA of the logarithm of WFOM fluorescence data (e.g. GCaMP or FAD) will yield two primary temporal components that closely resemble the fluorescence response and the haemodynamic confound. The spatial representation of these temporal components should be carefully examined to ensure that they share features with expected neural and haemodynamic components (e.g. the latter showing vessel structures). Ideally, components should be compared to simultaneously acquired diffuse-reflectance data to ensure a spatio-temporal likeness. In this case, the principal component time course found to represent haemodynamics can be multiplied by its spatial representation and subtracted from the (logarithmic) fluorescence dataset and the exponent then taken [19].

This methodology works optimally for short datasets capturing a single, simple spatio-temporal event such as a response to a localized stimulus. In more complex or longer duration data, where multiple fluorescence events take place in different regions, components will become much more difficult to separate reliably and blind-source techniques could remove important information from the dataset. In general, great care should be taken when using non-supervised algorithms such as PCA for this purpose, since even in the simplest case, the order of principle components can vary from one run to the next (e.g. haemodynamic could be first, second or third).

Performance of PCA (and regression techniques) can be improved with reduction of noise in the data, for example, by registering motion in each trial, averaging across trials and low-pass filtering (at approx. 3–5 Hz) to remove physiological noises such as heart rate and breathing rate.

## 4. Material and methods

### (a) Instrumentation

All data shown here was acquired using an LED-based WFOM system as described in [24,83] and depicted in figure 2. An Andor iXon 897 EMCCD camera was used to acquire all images shown, except figure 5, which used the Dalsa 1M60. In recent work, we have also used the Andor Zyla SCMOS camera to get similar results to the iXon. Simultaneous reflectance and fluorescence measurements are achieved by strobing illumination between up to four different colour LEDs using a microcontroller monitoring the camera's ‘expose’ signal. This approach allows precise control of the LED illumination period, differential control of their intensities, and allows the camera to run at its fastest free-running speed. We have made this software open source and available online [82]. Each high-power LED is mounted with a suitable band-pass filter to define a narrow spectral band and prevent leakage of excitation light (spectra shown in figure 4*g*). LEDs are combined using dichroics to yield a single beam that can be positioned to illuminate the brain directly. To achieve interlaced imaging of haemodynamics and GCaMP fluorescence, a 500 nm long-pass filter is positioned in front of the camera. This means that under (brighter) 480 nm excitation, fluorescence is detected, while during 530 and 630 nm illumination, reflectance signals are detected for spectral conversion to haemodynamics. This powerful strategy can be further expanded to include a near infrared laser diode within the strobe order to collect laser speckle images from which blood flow can also be calculated [51]. Images of different fields of view can be acquired using different lenses in front of the camera, e.g. Nikon AF Micro-NIKKOR 60 mm f/2.8D lens for the bilateral imaging shown in figure 1.

Other strategies for simultaneous imaging of fluorescence and reflectance include adding a second (or more) camera to the system, and splitting emitted light into a fluorescence and reflectance path. This approach can feasibly enable imaging of diffuse reflectance and the excitation wavelengths at the same time as fluorescence, and enable imaging at the cameras' highest frame rate. However, in addition to the cost of purchasing two cameras, synchronizing camera acquisition and exactly registering the images on both cameras can make correction analysis challenging.

### (b) Animal preparation

All animal procedures were reviewed and approved by the institutional animal care and use committee at Columbia University. Data shown in figure 5 was acquired in an intravenous alpha-chloralose anaesthetized female Sprague Dawley rat with an acute, unilateral glass cranial window placed after removal of approximately 3 × 4 mm region of skull and dura over the somatosensory cortex. The animal was ventilated via trachotomy. Full preparation details are provided in [11].

All other figures show data acquired in an awake, behaving transgenic mouse with a ‘chronic’ bilateral cranial window. (C57BL/6J-Tg(Thy1-GCaMP6f)GP5.17Dkim/J, strain purchased from Jackson Labs and bred in-house) [19]. Mice were anaesthetized with isoflurane, homeothermically maintained and monitored using pulse oximetry. In a fully aseptic environment, the scalp over the craniotomy area was resected. Skull thinning was then performed with constant saline irrigation, avoiding excessive drilling of the sutures but ensuring maximal removal of skull vessels or opaque skull. The thinning area can be extended beyond the bound of bregma and lambda, towards the frontal area for visualizing motor cortex or occipital area for cerebellum as needed. Once drilled to translucency, the dry surface of the skull was coated with a thin layer of cyanoacrylate (gel-type Loctite^®^ Super Glue) which serves to index-match the rough surface of the skull, prevent bone re-growth and provide mechanical protection [83]. The skin around the thinned skull preparation was sealed using cyanoacrylate tissue adhesive (e.g. 3M™ Vetbond™ Tissue Adhesive). A custom-made head plate made from laser-cut acrylic plastic was then used to enable immobilization (illustrated in figure 1) [84]. This approach enables the rapid manufacture of a custom-fit, light-weight, unobstrusive head plate for each mouse. The design encircles the bilaterally exposed cortex and is glued to the skull using more gel-type cyanoacrylate. To protect the thinned skull during recovery, a layer of Kwik-Sil™, a two-part, rapid cure silicone rubber is placed over the window and can be peeled-off for imaging (and then renewed following imaging). Post-operative care must ensure that animals are properly hydrated, have adequate pain relief (e.g. pre and post-operative buprenorphine) and are in an environment where they cannot catch or damage their head plates.

Training and handling of animals to perform behavioural tasks can be performed before surgery (without head-fixing) and following post-operative recovery. After recovery, the animal can be imaged repeatedly on subsequent days, enabling longitudinal studies of learning, development or disease progression without the confounds of anaesthesia affecting physiology and cognitive function. In our case, the animal was imaged while free to run on a horizontal ‘saucer wheel’ during imaging and tactile whisker stimulation (using a stepper motor actuator). The animal's running and behaviour was monitored throughout using a small camera. Data shown were averaged over repeated presentations of the same stimulus. However, the system described above also has sufficient signal to noise to record responses to single trials, or spontaneous neural and haemodynamic activity in the ‘resting’ brain [19].

## 5. Summary

Here, we have provided an overview of the basic optical theory of wide-field optical imaging methods for mapping the exposed cortex during functional imaging. Principles governing the spatial sensitivity, resolution and quantification limitations in haemodynamic imaging were presented, along with resources for standardization of analysis methods between researchers using the technique. Fluorescence WFOM in the context of emerging genetically encoded indicators of neural activity was described, with emphasis on the importance of correcting recordings for haemodynamic cross-talk. Theory and methods for cross-talk correction were presented and demonstrated.

## Supplementary Material

Appendix A & B

## Data accessibility

Datasets and methods required for analysis shown in this article have been uploaded as part of the electronic supplementary material. *In-vivo* imaging datasets will be provided by the corresponding author upon request.
