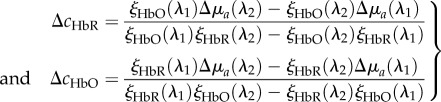# Correction to ‘Wide-field optical mapping of neural activity and brain haemodynamics: considerations and novel approaches’

**DOI:** 10.1098/rstb.2016.0539

**Published:** 2017-02-19

**Authors:** Ying Ma, Mohammed A. Shaik, Sharon H. Kim, Mariel G. Kozberg, David N. Thibodeaux, Hanzhi T. Zhao, Hang Yu, Elizabeth M. C. Hillman

*Phil. Trans. R. Soc. B* **371**, 20150360 (2016; Published 29 August 2016) (doi:10.1098/rstb.2015.0360)

After publication, a mistake was identified in equation (2.7); the denominators in the equation should have a minus sign rather than a plus. The corrected equation is provided here.
2.7