# Supplementary material for: Wide-field optical mapping of neural activity and brain haemodynamics: considerations and novel approaches
Source: Philos Trans R Soc Lond B Biol Sci. 2016 Oct 5;371(1705):20150360. doi: 10.1098/rstb.2015.0360 (PMC5003860; doi:10.1098/rstb.2015.0360)
Supplement: Appendix A & B [file rstb20150360supp1.pdf]

High-speed, wide-field optical mapping (WFOM) of neural activity and brain haemodynamics: Considerations and novel approaches

Appendix A

Tabulated Molar Extinction Coefficient for Hemoglobin in Water

These values for the molar extinction coefficient ε were compiled by Scott Prahl (Oregon Tech) [1] using data from:

W. B. Gratzer, Med. Res. Council Labs, Holly Hill, London
N. Kollias, Wellman Laboratories, Harvard Medical School, Boston

To convert this data to absorbance A, multiply the molar extinction coefficient ε [units: cm^{-1} M^{-1} where M = mol/L] by the molar concentration C [units: M] and the pathlength X [units: cm].

$$A = \varepsilon C X$$

If c is the concentration in [g/L], absorbance A is given by:

$$A = \frac{\varepsilon c X}{64{,}500}$$

where 64,500 is the gram molecular weight [units: g/mol] of hemoglobin.

To convert the molar extinction coefficient ε to absorption coefficient μ_a in [cm^{-1}], multiply by 2.303, the concentration in [g/L] and divide by the gram molecular weight of hemoglobin:

$$\mu_a = \frac{2.303\varepsilon c}{64{,}500}$$

A typical value of c for hemoglobin in whole blood is c = 150 g/liter.

Tabulated molar extinction coefficient ε for oxy and deoxyhemoglobin (HbO_2 and HbR):

Wavelength (nm)	ε $Hb0_2$ (cm^{-1} M^{-1})	ε HbR (cm^{-1} M^{-1})
250	106112	112736
252	105552	112736
254	107660	112736
256	109788	113824
258	112944	115040
260	116376	116296
262	120188	117564
264	124412	118876
266	128696	120208
268	133064	121544

270	136068	122880
272	137232	123096
274	138408	121952
276	137424	120808
278	135820	119840
280	131936	118872
282	127720	117628
284	122280	114820
286	116508	112008
288	108484	107140
290	104752	98364
292	98936	91636
294	88136	85820
296	79316	77100
298	70884	69444
300	65972	64440
302	63208	61300
304	61952	58828
306	62352	56908
308	62856	57620
310	63352	59156
312	65972	62248
314	69016	65344
316	72404	68312
318	75536	71208
320	78752	74508
322	82256	78284
324	85972	82060
326	89796	85592
328	93768	88516
330	97512	90856
332	100964	93192
334	103504	95532
336	104968	99792
338	106452	104476
340	107884	108472
342	109060	110996
344	110092	113524
346	109032	116052
348	107984	118752
350	106576	122092
352	105040	125436
354	103696	128776
356	101568	132120
358	97828	133632
360	94744	134940

362	92248	136044
364	89836	136972
366	88484	137900
368	87512	138856
370	88176	139968
372	91592	141084
374	95140	142196
376	98936	143312
378	103432	144424
380	109564	145232
382	116968	145232
384	125420	148668
386	135132	153908
388	148100	159544
390	167748	167780
392	189740	180004
394	212060	191540
396	231612	202124
398	248404	212712
400	266232	223296
402	284224	236188
404	308716	253368
406	354208	270548
408	422320	287356
410	466840	303956
412	500200	321344
414	524280	342596
416	521880	363848
418	515520	385680
420	480360	407560
422	431880	429880
424	376236	461200
426	326032	481840
428	283112	500840
430	246072	528600
432	214120	552160
434	165332	552160
436	132820	547040
438	119140	501560
440	102580	413280
442	92780	363240
444	81444	282724
446	76324	237224
448	67044	173320
450	62816	103292
452	58864	62640

454	53552	36170
456	49496	30698.8
458	47496	25886.4
460	44480	23388.8
462	41320	20891.2
464	39807.2	19260.8
466	37073.2	18142.4
468	34870.8	17025.6
470	33209.2	16156.4
472	31620	15310
474	30113.6	15048.4
476	28850.8	14792.8
478	27718	14657.2
480	26629.2	14550
482	25701.6	14881.2
484	25180.4	15212.4
486	24669.6	15543.6
488	24174.8	15898
490	23684.4	16684
492	23086.8	17469.6
494	22457.6	18255.6
496	21850.4	19041.2
498	21260	19891.2
500	20932.8	20862
502	20596.4	21832.8
504	20418	22803.6
506	19946	23774.4
508	19996	24745.2
510	20035.2	25773.6
512	20150.4	26936.8
514	20429.2	28100
516	21001.6	29263.2
518	22509.6	30426.4
520	24202.4	31589.6
522	26450.4	32851.2
524	29269.2	34397.6
526	32496.4	35944
528	35990	37490
530	39956.8	39036.4
532	43876	40584
534	46924	42088
536	49752	43592
538	51712	45092
540	53236	46592
542	53292	48148
544	52096	49708

546	49868	51268
548	46660	52496
550	43016	53412
552	39675.2	54080
554	36815.2	54520
556	34476.8	54540
558	33456	54164
560	32613.2	53788
562	32620	52276
564	33915.6	50572
566	36495.2	48828
568	40172	46948
570	44496	45072
572	49172	43340
574	53308	41716
576	55540	40092
578	54728	38467.6
580	50104	37020
582	43304	35676.4
584	34639.6	34332.8
586	26600.4	32851.6
588	19763.2	31075.2
590	14400.8	28324.4
592	10468.4	25470
594	7678.8	22574.8
596	5683.6	19800
598	4504.4	17058.4
600	3200	14677.2
602	2664	13622.4
604	2128	12567.6
606	1789.2	11513.2
608	1647.6	10477.6
610	1506	9443.6
612	1364.4	8591.2
614	1222.8	7762
616	1110	7344.8
618	1026	6927.2
620	942	6509.6
622	858	6193.2
624	774	5906.8
626	707.6	5620
628	658.8	5366.8
630	610	5148.8
632	561.2	4930.8
634	512.4	4730.8
636	478.8	4602.4

638	460.4	4473.6
640	442	4345.2
642	423.6	4216.8
644	405.2	4088.4
646	390.4	3965.08
648	379.2	3857.6
650	368	3750.12
652	356.8	3642.64
654	345.6	3535.16
656	335.2	3427.68
658	325.6	3320.2
660	319.6	3226.56
662	314	3140.28
664	308.4	3053.96
666	302.8	2967.68
668	298	2881.4
670	294	2795.12
672	290	2708.84
674	285.6	2627.64
676	282	2554.4
678	279.2	2481.16
680	277.6	2407.92
682	276	2334.68
684	274.4	2261.48
686	272.8	2188.24
688	274.4	2115
690	276	2051.96
692	277.6	2000.48
694	279.2	1949.04
696	282	1897.56
698	286	1846.08
700	290	1794.28
702	294	1741
704	298	1687.76
706	302.8	1634.48
708	308.4	1583.52
710	314	1540.48
712	319.6	1497.4
714	325.2	1454.36
716	332	1411.32
718	340	1368.28
720	348	1325.88
722	356	1285.16
724	364	1244.44
726	372.4	1203.68
728	381.2	1152.8

730	390	1102.2
732	398.8	1102.2
734	407.6	1102.2
736	418.8	1101.76
738	432.4	1100.48
740	446	1115.88
742	459.6	1161.64
744	473.2	1207.4
746	487.6	1266.04
748	502.8	1333.24
750	518	1405.24
752	533.2	1515.32
754	548.4	1541.76
756	562	1560.48
758	574	1560.48
760	586	1548.52
762	598	1508.44
764	610	1459.56
766	622.8	1410.52
768	636.4	1361.32
770	650	1311.88
772	663.6	1262.44
774	677.2	1213
776	689.2	1163.56
778	699.6	1114.8
780	710	1075.44
782	720.4	1036.08
784	730.8	996.72
786	740	957.36
788	748	921.8
790	756	890.8
792	764	859.8
794	772	828.8
796	786.4	802.96
798	807.2	782.36
800	816	761.72
802	828	743.84
804	836	737.08
806	844	730.28
808	856	723.52
810	864	717.08
812	872	711.84
814	880	706.6
816	887.2	701.32
818	901.6	696.08
820	916	693.76

822	930.4	693.6
824	944.8	693.48
826	956.4	693.32
828	965.2	693.2
830	974	693.04
832	982.8	692.92
834	991.6	692.76
836	1001.2	692.64
838	1011.6	692.48
840	1022	692.36
842	1032.4	692.2
844	1042.8	691.96
846	1050	691.76
848	1054	691.52
850	1058	691.32
852	1062	691.08
854	1066	690.88
856	1072.8	690.64
858	1082.4	692.44
860	1092	694.32
862	1101.6	696.2
864	1111.2	698.04
866	1118.4	699.92
868	1123.2	701.8
870	1128	705.84
872	1132.8	709.96
874	1137.6	714.08
876	1142.8	718.2
878	1148.4	722.32
880	1154	726.44
882	1159.6	729.84
884	1165.2	733.2
886	1170	736.6
888	1174	739.96
890	1178	743.6
892	1182	747.24
894	1186	750.88
896	1190	754.52
898	1194	758.16
900	1198	761.84
902	1202	765.04
904	1206	767.44
906	1209.2	769.8
908	1211.6	772.16
910	1214	774.56
912	1216.4	776.92

914	1218.8	778.4
916	1220.8	778.04
918	1222.4	777.72
920	1224	777.36
922	1225.6	777.04
924	1227.2	776.64
926	1226.8	772.36
928	1224.4	768.08
930	1222	763.84
932	1219.6	752.28
934	1217.2	737.56
936	1215.6	722.88
938	1214.8	708.16
940	1214	693.44
942	1213.2	678.72
944	1212.4	660.52
946	1210.4	641.08
948	1207.2	621.64
950	1204	602.24
952	1200.8	583.4
954	1197.6	568.92
956	1194	554.48
958	1190	540.04
960	1186	525.56
962	1182	511.12
964	1178	495.36
966	1173.2	473.32
968	1167.6	451.32
970	1162	429.32
972	1156.4	415.28
974	1150.8	402.28
976	1144	389.288
978	1136	374.944
980	1128	359.656
982	1120	344.372
984	1112	329.084
986	1102.4	313.796
988	1091.2	298.508
990	1080	283.22
992	1068.8	267.932
994	1057.6	252.648
996	1046.4	237.36
998	1035.2	222.072
1000	1024	206.784

Appendix B

Scattering and absorption properties

Values for the scattering properties of rat brain used in simulations shown here were taken from measurements presented in the thesis of Peter Van der Zee [2] with the relevant figure reproduced below:

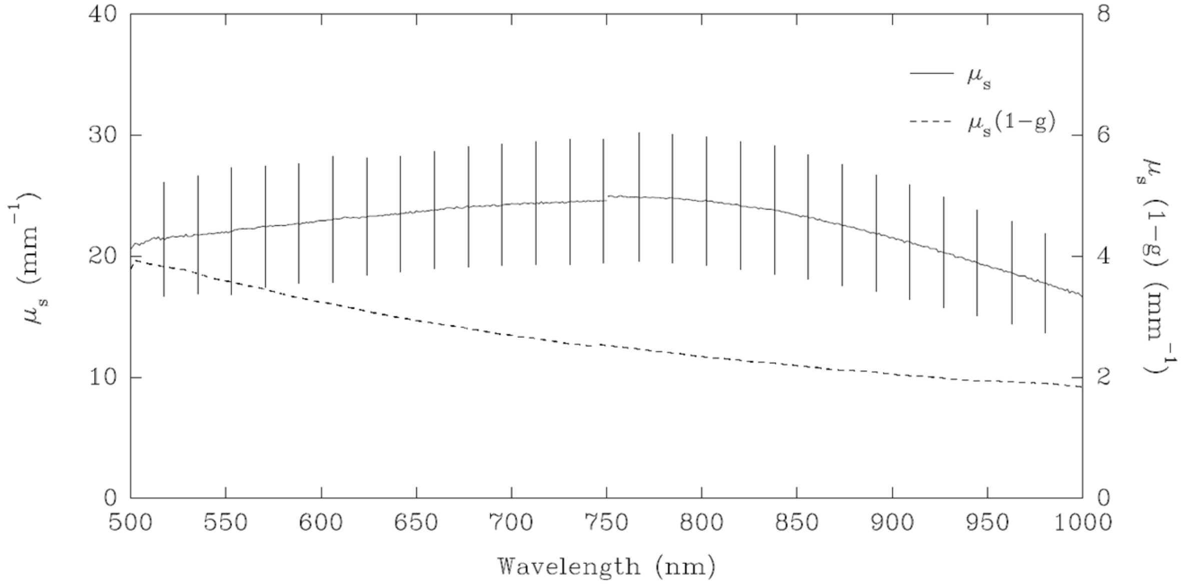

Supp Figure 1 Scattering values taken from blood free rat brain [2] and used in models provided here.

Here, μ_s described the scattering coefficient (1/scattering length), while $\mu_s' = \mu_s(1\text{-}g)$ is the reduced scattering coefficient which denotes the effective scattering coefficient if all interactions resulted in completely isotropic scattering. g is the anisotropy factor which describes the directionality of each scattering event (given by the shape, size and composition of the scatterers). g = 0 represents isotropic scattering such that $\mu_s' = \mu_s$. The plots above allow derivation of values of g for brain tissue from μ_s' as used in our models:

Wavelength	μ_s (mm^{-1})	g	μ_s' (mm^{-1})	μ_a (mm^{-1})
488 nm	21	0.8	4.2	0.33
530 nm	21	0.82	3.78	0.55
630 nm	24	0.87	3.12	0.024

Absorption values are derived from values in appendix A and assuming 3% blood in tissue and 75% average oxygen saturation.

Monte Carlo modeling

Monte Carlo modeling simulates light propagation by repeatedly launching photons into a modeled medium with pre-defined optical properties and a specific illumination and detection geometry. The probability and direction of scattering events is given by the values of μ_s and g. Each photon is tracked along its trajectory, recording its cumulative distance travelled, and the locations it visited along its path

as well as the location and angle at which it exits the tissue. Reciprocity is a rule that is often used in Monte Carlo models, and assumes that light propagation in one direction is equivalent to propagation in the reverse direction. Thus, to simulate, for example, illumination of a large area of a scattering surface and detection of light emerging at a point, one can simulate light entering at a point, and emerging from any point on the same surface. Depending on the sample volume, 10,000 to 10^9 photon launches will be needed to provide a good representation of light distribution. Two types of modeling were used here.

1) For pathlength calculation

Using reciprocity, we simulate light entering the tissue with a beam whose NA matches our camera's lens (here, 0.12). The Monte Carlo is run 'white' which means only scattering probability is considered. 10^9 photons that entered were tracked, and the location of exit, direction of exit and cumulative pathlength for each is recorded. To convert this data to pathlength, first, only photons exiting the surface with an angle normal to the surface were considered (analogous to unidirectional illumination of the surface with LED light). Second, the pathlength of each photon was then converted to a weight, representing its likelihood of existing based on the absorption coefficient of the medium it traveled through using the Beer Lambert law (Eq 1). The total average pathlength of light is then calculated by summing each photon's weight multiplied by its pathlength:

$$X_{DR}(\lambda) = \sum_{n=1}^{N} x_n \, e^{-\mu_a(\lambda) x_n}$$

Supp Eq 1

Using the values for scattering tabulated above, scattering properties have a relatively small effect of pathlength compared to the effects of absorption as shown below in Supp Figure 2. Here, pathlength was calculated using a wavelength-specific scattering model, and then accounting for corresponding wavelength-dependent absorption using Supp Eq 1. Given the similarity of these pathlength traces, the data tabulated below utilizes the scattering properties assumed for 630 nm light.

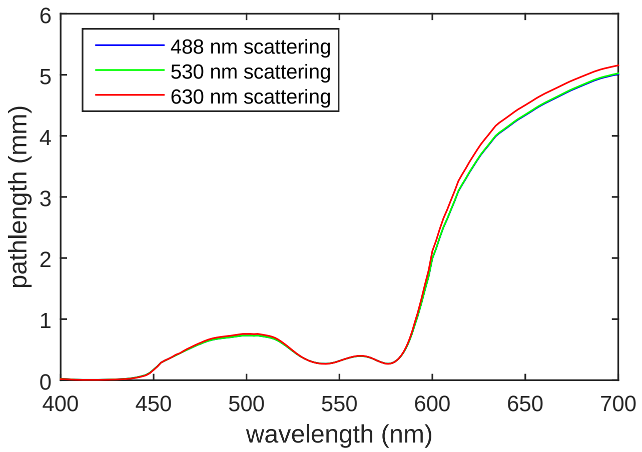

Supp Figure 2. Plots of average pathlength calculated using scattering properties at three different wavelengths

2) Modeling depth sensitivity

The plots shown in Figure 4 of the main paper were generated using a different form of Monte Carlo modeling. Using the parameters tabulated above, including absorption, the model was run to simulate light entering the tissue at an NA of 0.12. In this case, the amount of light reaching each point within the 3D volume was added up (based on the cumulative decrease in probability of existing, given the photon's pathlength through the absorbing medium). At each point, the average direction of propagation of each visiting photon was also recorded. Given the cylindrical symmetry of the geometry, and to reduce both the time needed to run the model, and the size of the data produced, data was logged as a 2D distribution of intensities and propagation angles across r (radius from the source) and z (depth).

To estimate the average chance of a photon entering the tissue, visiting a point r,z, and then exiting the tissue again and reaching the camera, we can again use reciprocity. The probability of light incident at the surface reaching a given depth in the medium is equivalent to the sum of all probabilities in our model across x and y for a given depth z (equivalent to translating and summing the source over the whole uniform field of view). The probability of a photon emerging from a point in the tissue and exiting at the pixel of interest at an angle acceptable to the camera lens is equal to the intensity of the light reaching any point in the medium after entering at that pixel location with an angle less than or equal to the detection NA. The combined probability is then the product of these two distributions: the probability of reaching a point r,z and the probability of making it back out again. This probability equates to the amount of change in a measurement that would occur for a given change in the absorption coefficient at that location (for small changes that can be assumed linear). Given that light is directional for reflectance imaging across these relatively small distances (<1 mm) one additional consideration was that the direction of propagation of the incoming and outgoing light was accounted for via the dot product of the light direction vector. A similar strategy is described here [3-5].

Tabulated estimated pathlength values for in-vivo rat / mouse brain with wide-field illumination and detection:

Wavelength (nm)	Estimated average pathlength X (mm)
400	0.022148
402	0.01959
404	0.016739
406	0.013091
408	0.009544
410	0.007862
412	0.006824
414	0.00613
416	0.006034

418	0.006016
420	0.006615
422	0.007661
424	0.009103
426	0.010869
428	0.012758
430	0.014514
432	0.016324
434	0.021095
436	0.025688
438	0.030731
440	0.042163
442	0.051656
444	0.071701
446	0.087201
448	0.121648
450	0.173374
452	0.225728
454	0.288434
456	0.322998
458	0.347592
460	0.377372
462	0.412918
464	0.433427
466	0.467785
468	0.499849
470	0.526691
472	0.554803
474	0.580816
476	0.604492
478	0.626572
480	0.64898
482	0.666149
484	0.67508
486	0.683987
488	0.692555
490	0.697841
492	0.705741
494	0.714562
496	0.723036
498	0.730719
500	0.73081

502	0.731133
504	0.727479
506	0.731214
508	0.721874
510	0.712537
512	0.700563
514	0.685225
516	0.664134
518	0.625646
520	0.58763
522	0.544338
524	0.49737
526	0.45224
528	0.410981
530	0.371713
532	0.338528
534	0.315274
536	0.295698
538	0.282444
540	0.272231
542	0.269437
544	0.272728
546	0.281333
548	0.296392
550	0.316101
552	0.336472
554	0.355977
556	0.374246
558	0.383791
560	0.392168
562	0.396586
564	0.390147
566	0.373314
568	0.3498
570	0.324347
572	0.299444
574	0.280016
576	0.271559
578	0.278296
580	0.306261
582	0.354421
584	0.434922

586	0.542064
588	0.677941
590	0.847256
592	1.040145
594	1.252074
596	1.481586
598	1.702211
600	1.998288
602	2.156612
604	2.342275
606	2.51064
608	2.645002
610	2.794747
612	2.940892
614	3.100511
616	3.205588
618	3.305013
620	3.410977
622	3.506547
624	3.602268
626	3.693374
628	3.771878
630	3.846483
632	3.924145
634	4.000972
636	4.053497
638	4.096681
640	4.140704
642	4.185688
644	4.231663
646	4.274555
648	4.311313
650	4.348707
652	4.386754
654	4.425469
656	4.464193
658	4.502907
660	4.535122
662	4.565273
664	4.595836
666	4.626793
668	4.657428

670	4.687721
672	4.718404
674	4.748249
676	4.775131
678	4.801533
680	4.82704
682	4.852812
684	4.878841
686	4.905157
688	4.928464
690	4.948468
692	4.964616
694	4.980853
696	4.995942
698	5.009848
700	5.023943

Supplemental Material references:

1. Prahl, S.A., *Online resource: http://omlc.ogi.edu/spectra/hemoglobin/summary.html*.
2. van-der-Zee, P., *Measurement and modelling of the optical properties of human tissue in the near infrared*, in *Department of Medical Physics and Bioengineering*. 1992, University College London: London. p. 313.
3. Hillman, E.M.C., D.A. Boas, A.M. Dale, and A.K. Dunn, *Laminar Optical Tomography: demonstration of millimeter-scale depth-resolved imaging in turbid media.* Opt. Lett., 2004. **29**(14): p. 1650-1652.
4. Hillman, E.M., A. Devor, M.B. Bouchard, A.K. Dunn, G.W. Krauss, J. Skoch, B.J. Bacskai, A.M. Dale, and D.A. Boas, *Depth-resolved optical imaging and microscopy of vascular compartment dynamics during somatosensory stimulation.* Neuroimage, 2007. **35**(1): p. 89-104.
5. Dunn, A.K. and D.A. Boas, *Transport-based image reconstruction in turbid media with small source–detector separations.* Opt. Lett., 2000. **25**(24): p. 1777-1779.
